# Surgicogenomics: The Role of Genetics in Deep Brain Stimulation in Parkinson’s Disease Patients

**DOI:** 10.3390/brainsci14080800

**Published:** 2024-08-09

**Authors:** Kallirhoe Kalinderi, Vasileios Papaliagkas, Liana Fidani

**Affiliations:** 1 Laboratory of Medical Biology-Genetics, School of Medicine, Faculty of Health Sciences, Aristotle University of Thessaloniki, 54124 Thessaloniki, Greece; sfidani@auth.gr; 2 Department of Biomedical Sciences, School of Health Sciences, International Hellenic University, 57400 Thessaloniki, Greece; vpapaliagkas@gmail.com

**Keywords:** Parkinson’s disease, surgicogenomics, gene, GBA, deep brain stimulation, cognitive decline, quality of life, parameters, motor improvement, G2019S LRRK2

## Abstract

Parkinson’s disease (PD) is the second-most common neurodegenerative disease, affecting 1% of people aged over 60. Currently, there is only symptomatic relief for PD patients, with levodopa being the gold standard of PD treatment. Deep brain stimulation (DBS) is a surgical option to treat PD patients. DBS improves motor functions and may also allow a significant reduction in dopaminergic medication. Important parameters for DBS outcomes are the disease duration, the age of disease onset, responsiveness to levodopa and cognitive or psychiatric comorbidities. Emerging data also highlight the need to carefully consider the genetic background in the preoperative assessment of PD patients who are candidates for DBS, as genetic factors may affect the effectiveness of DBS in these patients. This review article discusses the role of genetics in DBS for PD patients, in an attempt to better understand inter-individual variability in DBS response, control of motor PD symptoms and appearance of non-motor symptoms, especially cognitive decline.

## 1. Introduction

Parkinson’s disease (PD) is the second-most common neurodegenerative disease, after Alzheimer’s disease, with its prevalence continually increasing. Nowadays it affects 1–2% of the population over 65, and this percentage rises to 3–5% at ages beyond 85 years [1]. By 2040, it is expected that almost 12 million people will be diagnosed with PD. The main characteristics of the disease are a resting tremor, bradykinesia, rigidity, postural instability and freezing episodes, usually accompanied by a variety of non-motor features, such as cognitive decline, behavioral symptoms or sleep disturbances [2,3]. Currently, there is only symptomatic relief for patients, with levodopa being the gold standard of PD treatment, although, during the last few years, novel agents are being used with promising results [4]. 

Deep brain stimulation (DBS) is a surgical option to treat PD patients by using implanted electrodes and high-frequency electrical stimulation of selected targets, which are usually the subthalamic nucleus (STN), the globus pallidus internus (GPi) and the ventralis intermediate nucleus (Vim) of the thalamus. This neurosurgical procedure intervenes and restores the abnormal information that is transferred through the cortico-basal ganglia loop in PD, improving motor and non-motor symptomatology and quality of life of PD patients [5]. Ideal clinical indications have been proposed to be PD duration > 5 years (>4 years in early stimulation), age < 75 years, motor improvement > 30% with levodopa, significant motor fluctuations and dyskinesia and a medication-resistant tremor [6]. Thus, among the important parameters for the success of this operation are the disease duration, the age of disease onset, responsiveness to levodopa as well as the presence of cognitive or psychiatric comorbidities; favorable outcomes are usually seen in patients of a young age, with a short disease duration, no levodopa-resistant axial signs and normal cognitive function and psychiatric symptoms [5]. DBS may be contraindicated in cases of dementia, hallucinations, uncontrolled depression, severe postural and gait disabilities, increased brain atrophy or intracranial lesions affecting surgical approach, severe systemic disease or atypical parkinsonism [6].

In the era of precision medicine, decoding patients’ genetic background is fundamental in order to deeply understand the clinical heterogeneity of PD and the variability in responses to treatment. Emerging data suggest that genetic factors may affect the DBS outcomes of PD patients. This review article focuses on the genes that have so far been examined in respect to DBS in PD patients, in an attempt to better understand inter-individual variability in DBS response, control of motor PD symptoms and appearance of non-motor symptoms, especially cognitive decline.

## 2. Methods

For this narrative review article, we searched PubMed and Scopus databases for peer-reviewed research, review articles and meta-analyses regarding the role of genetics in DBS in PD patients, with no time restrictions. The keywords used were autosomal dominant Parkinson’s disease, autosomal recessive Parkinson’s disease, monogenic Parkinson’s disease, deep brain stimulation, Parkinson’s disease, parkinsonism, gene, genetic, mutation, polymorphism, surgicogenomics, motor symptoms, non-motor symptoms, co-morbidities. We also screened the references of the selected articles for possible additional articles in order to include most of the key recent evidence. Regarding the inclusion criteria, all studies were in the English language, performed on humans and referred to genetic PD patients who were offered DBS treatment. Studies were excluded in the case that the title and/or the abstract were not compatible with the aim of this narrative review.

## 3. Results

### 3.1. GBA

The glucocerebrosidase (GBA) gene is located on chromosome 1 (1q21) and encodes for the lysosomal enzyme glucocerebrosidase. Homozygous GBA mutations are associated with the lysosomal storage disorder called Gaucher’s disease; however, relatives of these patients have been observed to be at increased risk for PD [7,8]. More specifically, GBA variants have been found to increase the risk of PD 5–30-fold, depending on age, ethnicity and the studied mutations [9]; moreover, carriers of GBA mutations have been associated with an earlier age of disease onset, more rapid motor deterioration, earlier appearance of neuropsychiatric symptoms and approximately six-fold increased risk of developing dementia compared to non-carriers [9,10,11].

In the first study providing data on the therapeutic efficacy of long-term STN-DBS in PD due to heterozygous GBA mutations, among 98 PD patients treated with STN-DBS, three GBA mutation carriers (one GBA N370S and two GBA L444P) were detected and followed for up to 10 years. All GBA carriers showed cognitive impairment. Motor fluctuations and dyskinesias were well controlled postoperatively and the levodopa dose used was substantially decreased. Segmental and axial symptoms were improved in the first 4 years after surgery; however, 4 to 6 years from the operation an increase in axial motor impairment along with a decline in therapeutic efficacy in GBA carriers was observed. In parallel, cognitive decline was reported in all GBA carriers [12]. In another study, 94 patients who underwent DBS were examined for known PD mutations. Among them, 16 patients had at least one GBA mutation, most commonly GBA E326K. In 13 GBA carrier patients, the target for DBS was the STN, in two, the GPi and in one patient, the Vim. Postoperative assessments were performed after a 12-month period. The response did not differ significantly between GBA subgroups; however, in GBA patients, a steeper cognitive decline was observed in a 5-year follow-up after DBS [13]. In the study of Lythe et al., of the examined 34 PD DBS patients, 17 had GBA mutations and were matched to 17 non-carriers. No significant difference in motor function between GBA carriers and non-carriers was observed after surgery. Moreover, after a follow-up of 7.5 years, cognitive impairment appeared to be more frequent and severe in GBA patients compared to controls. Likewise, non-motor symptoms were also more severe, and quality of life was lower in GBA PD patients [14]. In a larger study of 208 PD patients, among which 25 had GBA mutations, improvement in motor function was observed in GBA mutation carriers compared to non-carriers after surgery, however, with no significant difference between the studied groups. Importantly, earlier cognitive decline was detected in GBA mutation carriers [15]. Results from reviews and meta-analyses also showed that carriers of GBA mutations displayed faster cognitive and functional decline after DBS [16,17]. Neri et al. also reported a patient carrying a frameshift deletion in the LRP10 gene (c.348_349delCA, p. Tyr116*) and the p. Leu444Pro GBA variant who underwent DBS with improvement in motor function, but rapid cognitive deterioration [18]. However, in a patient with G325R GBA mutation who was treated with STN-DBS, the cognitive and neurobehavioral performances remained stable over time and did not decline post-surgically [19]. In another case report, in a patient with Gaucher Disease Type 1-Associated PD, there was an eminent improvement in motor functions and quality of life after DBS, which persisted 3 years after the operation, with no cognitive decline reported in the follow-up tests [20]. In 2022, a multicenter study examined longitudinal cognitive results from 366 PD patients with or without GBA1 mutations and treated or not with STN-DBS. Improved motor function was observed in GBA mutation carriers after surgery with no significant difference between the GBA mutation carriers and non-carriers. There was a reduction in the levodopa dose used in the GBA carriers after surgery, as well. Interestingly, this retrospective study reported a more rapid cognitive decline in the group of PD-GBA1 treated with STN-DBS compared to non-GBA1 carriers treated with DBS, non-GBA1 carriers not treated with DBS and PD-GBA1 not treated with DBS. There was no difference in the rate of cognitive decline when comparing patients with mild versus severe GBA mutations, although the sample size was limited [21]. Interestingly, in a recent study that examined the combined effects of GBA1 mutations and STN-DBS on cognitive function, it was found that in GBA1 + DBS+ PD patients, the pattern of cognitive dysfunction was more severe [22]. In a large retrospective Italian cohort, 365 patients with PD were included, of whom 73 were carriers of GBA variants. Data after a 5-year follow-up could be retrieved from 173 PD patients, of whom 32 were GBA mutation carriers. After DBS, a significant motor improvement, as well as a reduction in fluctuations, dyskinesias and impulsive-compulsive disorders, was reported. Cognitive scores had deteriorated at a faster rate in GBA-PD at a 3-year follow-up, but the majority of GBA-PD had not developed dementia at a 5-year follow-up [23]. In a recent multi-scale meta-analysis on motor and non-motor outcomes of DBS in PD patients with different genetic mutations, GBA carriers appeared to be more prone to cognitive decline after DBS compared to the other genetic groups. The pattern of cognitive decline was different according to different GBA variants. Among GBA carriers, the patient with the mutation GBA: L444P/LRP10: Y116* had the worst cognitive outcome, whereas the best outcome was reported in a carrier of the L444P variant (GBA: L444P/LRRK2:G2019S). The cognitive function was also suggested to be influenced by the mutations in other genes [24] (Table 1). 

Whether there is an interaction between GBA status and the risk of subsequent cognitive decline after STN-DBS needs to be further clarified. Preoperative cognitive problems are a risk factor for accelerated postoperative cognitive decline, thus a careful selection of these patients is needed. The best target of surgical therapy suited for GBA1-PD patients is also under investigation. The overall benefit of DBS should also be considered regarding factors such as motor improvement, quality of life, side effects of anti-parkinsonian drugs and risk of non-motor symptoms including depression, anxiety or psychosis. Other factors affecting cognitive function such as operation time, type of anesthesia, anti-parkinsonian medication prescribed post-surgically and exercise are also some parameters that should be evaluated with caution. Thus, when considering a GBA1 patient, the potential benefits and DBS risks should be carefully considered and current data and gaps should be discussed with the patient. 

### 3.2. SNCA

The a-synuclein (SNCA) gene is located on chromosome 4q21.3-q22 and encodes for a-synuclein, a 140 amino-acid-soluble protein mainly expressed in the central nervous system, especially in presynaptic terminals. SNCA was the first gene implicated in the genetic etiology of PD. Various SNCA mutations including missense mutations and multiplications have been associated with autosomal dominant PD [25]. SNCA triplication, but also duplication, is especially associated with early-onset, rapidly progressive PD with increased risk of cognitive impairment.

Shimo et al. were the first to report a 41-year-old patient with a duplication of SNCA with no signs of dementia or depression who received bilateral STN-DBS; after the 4-year follow-up, there was an improvement in motor fluctuation and a reduction in the dose of the anti-parkinsonian drugs used [26]. As patients with a duplication of SNCA have a high probability of psychiatric problems [27], it is recommended to have a long-term follow-up after STN-DBS. In another case report, STN-DBS was performed on a 46-year-old female carrier of SNCA duplication. One year postoperatively, there was a reduction of 64% on the Unified Parkinson’s Disease Rating Scale (UPDRS) III in ‘‘off medication’’ and no peak-dose dyskinesias were reported. Postoperative cognitive decline was observed, especially in verbal fluency and attention shifting [28]. In a 26-year-old male PD patient with a mosaicism of SNCA duplication, GPi-DBS was successfully performed with improvement in motor features, no peak-dose dyskinesias, reduction in the dose of anti-parkinsonian drugs and no major adverse events [29]. The role of genetic variants in DBS outcomes was also addressed by a recent study in which 85 PD patients who were treated with STN-DBS were examined for SNCA rs356219 and rs356220 polymorphisms in the 3’ untranslated region of the gene. PD patients carrying these SNCA polymorphisms, especially homozygous carriers, had a better response to DBS regarding motor and axial symptomatology after two years of follow-up compared to wild type [30]. However, in another study, the SNCA rs356220 variant did not predict UPDRS III outcomes but was associated with improvement in quality of life [31]. Additional larger, multicenter and prospective studies examining these and other genetic polymorphisms are needed. Interestingly, the same researchers reported that preoperative motor and affective parameters can predict quality of life after STN-DBS surgery [32]. Moreover, in a multicenter retrospective study on PD patients with SNCA mutations that received bilateral STN-DBS, data for long-term outcomes were obtained, with sustained motor improvement in the patients with SNCA duplication and a significant decrease in the dose of levodopa in the patient with the SNCA missense c.158C.A (p.A53E) mutation. However, despite the benefit to motor fluctuation, this patient became wheelchair-bound due to progressed axial, cognitive and psychiatric symptoms 3.5 years postoperatively [33] (Table 2).

DBS could be considered in PD patients with SNCA mutations. Long-term efficacy should be further examined and factors such as cognitive function and psychiatric symptomatology should be carefully taken into consideration, as they seem to affect the outcome of DBS in PD patients with SNCA mutations.

### 3.3. LRRK2

The leucine-rich repeat kinase 2 (LRRK2) gene is located on chromosome 12q12. LRRK2 mutations have been associated with both familial and sporadic PD, with the most common variant being G2019S [34]. Patients with G2019S LRRK2 PD develop a similar phenotype to idiopathic PD, such as an asymmetrical resting tremor, bradykinesia, rigidity and good levodopa responsiveness. LRRK2-PD patients are also at risk for developing motor fluctuations, dyskinesias and dystonia [35].

Johansen et al. enrolled 630 consecutive patients with PD that were genetically screened; mutations in LRRK2, PRKN and PINK1 were detected. Among these patients, 60 received DBS and three out of 60 PD patients had the LRRK2 G2019S mutation. Patients with PD-associated mutation had a comparable clinical outcome to PD patients without known PD mutations after 3 years of follow-up [36]. Moreover, in another cohort, 94 patients who underwent DBS were screened for mutations in PD-associated genes. Five patients had the G2019S mutation and one of them had also the E326K GBA mutation. Similar clinical outcomes were observed in PD mutation carriers and non-carriers after STN-DBS [13]. Similarly, the LRRK2 G2019S mutation status did not affect the outcome of STN-DBS in PD patients in a study including 39 Jewish PD patients with bilateral STN-DBS, 13 of whom had the G2019S LRRK2 mutation; this result was also supported by the study of Fernandez et al. [37]. Greenbaum et al. reported significant improvement in UPDRS III (off medication, on stimulation) and reduction in the levodopa dose used after surgery; however, no significant difference was detected between mutation and non-mutation carriers [38]. In another study, in 69 patients who received STN-DBS, eight had the G2019S LRRK2 mutation and one the T2031S LRRK2 mutation. Motor symptoms, levodopa-induced complications and daily living activities improved significantly, although the clinical responses after STN-DBS were comparable in both groups; however, in two G2019S PD patients, a sustained beneficial effect was observed 9 and 10 years postoperatively. Regarding the patient with the T2031S mutation, psychotic complications, addiction to levodopa and mild cognitive decline were recorded 5 years after surgery [39]. No difference was observed in a study examining the rs1491923 polymorphism, either [30]. Moreover, in a recent study of 103 patients, 19 had LRRK2 mutations, 20 GBA mutations and 64 were non-carriers PD patients; no significant differences in motor outcomes were found between the studied groups, as well. Notably, in the GBA-PD patients, psychotic episodes and cognitive decline were more common [40]. On the other hand, Sayad et al. in a study of 27 Algerian PD STN-DBS patients reported that PD patients with the G2019S LRRK2 mutation had a better surgical response compared to non-carriers, with greater improvement in UPDRS III in mutation carriers when switched from the off-medication, off-stimulation condition to off-medication, on-stimulation condition, and greater improvement in functional status in mutation carriers when stimulated without medications. The G2019S mutation was present in 55.6% of PD patients who received DBS stimulation [41]. Good motor response was also detected in another patient with the G2019S mutation; however, levodopa-related dystonia was observed [42]. In addition, in the systematic review of Artusi et al., LRRK2 mutation carriers showed sustained improvements only in UPDRS-IV [17]. In a recent study, the profile of PD patients with LRRK2 mutations that undergo DBS was recorded; among this group of patients, younger age of onset, longer disease duration and dyskinesia were commonly present. After 2 years of follow-up, these patients had a slightly slower rate of motor progression compared to idiopathic PD-DBS patients [43]. 

Regarding other LRRK2 mutations, a positive response to DBS has been reported for Y1699C or R793M mutations [44,45], whereas in the Spanish population, the response of R1441G mutation carriers has been worse compared to non-carriers—a result that was replicated in a recent systematic review [16,46]. Severe motor psychiatric complications were also observed in a patient carrying a double LRRK2 R1441G and G2385R mutation [47]. Furthermore, in a study of the Chinese population, where the G2385R is a common variant, a significant improvement in UPDRS II and III (off-medication) scores after surgery was observed, although with no significant difference between the studied groups [48]. Thus, the benefit of DBS might be restricted to some LRRK2 patients, depending on the mutation they carry (Table 3).

Additional studies are required to better understand the response of PD patients carrying the G2019S or other LRRK2 mutations to DBS, as the presence of different mutations in this gene appears to affect the results of the operation.

### 3.4. Parkin

Parkin is located on chromosome 6q25.2–27. Mutations in parkin are the most common cause of autosomal recessive early-onset PD. PD Patients with parkin mutations are characterized by slower disease progression and a better response to lower doses of levodopa compared to idiopathic PD patients. Other characteristics are the early appearance of dystonia and dyskinesia, and postural decline [25]. These patients tend also to develop mood disorders without executive dysfunction or cognitive impairment [49].

In the study of Lohman et al., studying the effect of DBS, seven PD patients with one parkin mutation, seven patients with two parkin mutations and 39 patients without parkin mutations were included. Motor outcomes were similar in the mutation carriers and non-mutation carriers after surgery and there was no significant change in cognitive function. After 2 years of follow-up, the administered levodopa doses were significantly lower in patients with two parkin mutations compared to patients without mutations and there was also a trend for PD patients with one parkin mutation to receive lower levodopa doses, albeit without reaching statistical significance [50]. In the study of Moro et al., 3–12 months after surgery, a mean improvement in the UPDRS III (off medication) was seen, and the improvement in motor function was found to be sustained for 3–6 years after surgery. In the long-term follow-up, a similar degree of clinical improvement was observed in mutation carriers compared to non-mutation carriers [51]. Other studies showed that there was no significant difference in the degree of improvement achieved with DBS, between mutation-negative PD patients and PD patients with parkin mutations [13,36,52,53].

Interestingly, in a case of an individual with juvenile parkinsonism due to homozygous deletion of exon 3 in the parkin gene after STN-DBS, there was significant motor improvement and a reduction in the levodopa-equivalent daily dose medication by 66%, as well as complete resolution of severe dyskinesias [54]. In another case report of juvenile-onset parkinsonism with both parkin and PINK1 mutations that was treated with STN-DBS at 45 years after disease onset, eminent improvement in PD symptoms was observed after surgery [55]. The benefit of DBS in PD patients with long duration and parkin mutations was also confirmed by the study of Lefaucheur et al., who support that whatever the disease duration is, stimulation of the subthalamic nucleus improves parkin-associated PD [56]. Successful treatment of juvenile PD with bilateral STN-DBS in a 14-year-old pediatric PD patient with parkin mutation has also been reported, suggesting the possible effectiveness of DBS in the pediatric population with parkin mutations [57]. Importantly, in a recent study in a 39-year-old PD patient with parkin mutation who received DBS, a significant improvement in motor symptoms and fluctuations was reported that were well controlled, after a 15-year follow-up evaluation [58]. A recent study also suggested that sex hormones may have a substantial impact on the function of the dopaminergic system; a 21-year-old woman, with a homozygous c.G859A parkin mutation was treated with STN-DBS with eminent clinical improvement after the operation but the patient soon reported more “off” periods with painful dystonia, during the luteal phase of her menstrual cycle. Levels of progesterone were found to influence beta band activity in the STN, suggesting a possible connection between sex hormones and motor fluctuations [59] (Table 4).

Current data support that DBS appears to be effective in early-onset PD and particularly in PD patients with PRKN mutations. Although a number of case reports suggest that STN-DBS may improve motor symptoms in autosomal recessive juvenile parkinsonism (ARJP), the therapeutic effects of STN-DBS in these patients remain to be further investigated. The favorable outcome of DBS on juvenile parkinsonism in pediatric patients has also been reported; however, it requires confirmation in larger pediatric cohorts with longer follow-up. Moreover, the type of PRKN mutations could influence the result of DBS and should also be taken into consideration. Longer follow-up and additional observations are needed to draw more definite conclusions. 

### 3.5. PINK1

The PINK1 gene is located on chromosome 1p35-p36. Mutations in this gene are found in 2–4% of early-onset PD (EOPD) and 4–9% of EOPD in Caucasian and Asian populations, respectively. The clinical characteristics of these patients are indistinguishable from those of other EOPD patients, with generally good levodopa response and slow disease progression. Some PD patients may develop dementia at a later stage of the disease [25].

A case of a patient with homozygous 619C>T-p. (Arg207*) PINK1 mutation who received STN-DBS has been described: initially, substantial motor improvement was observed; however, in the follow-up, dyskinesias, freezing of gait and a sub-continuous tremor were eminent, although with good control of motor fluctuations; better quality of life compared to the presurgical status was also reported [60]. More et al. suggested that patients with PINK1 mutations may benefit from STN-DBS; however, there was no significant difference in motor symptomatology compared to non-mutation carriers [51]. Interestingly, in 2017, Borellini et al. reported a Filipino woman with homozygous L347P PINK1 mutation with severe lower limb dystonia, gait abnormalities and dyskinesias who was treated with STN-DBS. Although a transient benefit was initially reported, the patient after a 4-year follow-up was not responsive to medical therapy and stimulation, and was unable to walk, mainly due to worsening of dystonia [61,62] (Table 5).

Additional studies including PD carriers of PINK1 mutations are needed in order to elucidate the role of STN-DBS for these patients. Limited data support that PINK1 mutation carriers show a transient therapeutic benefit after surgery; however, in the long-term follow-up they developed axial symptoms and dyskinesia and were almost unresponsive to drug therapy and neurostimulation. Long-term multicenter studies are needed to understand the impact of DBS on PINK1 mutation carriers.

### 3.6. VPS35

The vacuolar protein sorting 35 homolog (VPS35) gene is located on chromosome 16 and has recently been found as a cause of late-onset autosomal dominant PD, with the VPS35 D620N mutation being the most frequent mutation. The clinical characteristics of PD patients with the VPS35 mutation are similar to idiopathic PD, with asymmetry at onset, slow progression of the disease and levodopa responsiveness [25].

A limited number of PD patients with the VPS35 D620N mutation have been treated with STN-DBS, with good response after a follow-up of up to 8 years [63,64] (Table 6).

Additional studies are expected to increase our knowledge regarding the usefulness of DBS for VPS35 mutation carriers.

## 4. Discussion

In the era of precision medicine, a patient’s genetic background is important to understanding the clinical heterogeneity of PD and the varying responses to treatment. DBS is a surgical option to treat PD patients. Previously, crucial parameters for appropriate patient selection for this surgical procedure involved disease subtype, age of disease onset, responsiveness to levodopa and cognitive or psychiatric issues. Recent evidence highlights the importance of genetics to DBS in PD patients. DBS outcomes differ according to the genetic subtype, with significant clinical improvement in some cases. Genes like GBA, SNCA, LRRK2 and PRKN are the most commonly studied. Mutations in these genes have been detected in up to 29% of PD patients who receive DBS. In the short-term follow-up, most PD patients carrying PRKN, LRRK2 (except for R144G) and GBA mutations had positive DBS outcomes, with marked or satisfactory responses. In the intermediate follow-up, most patients with PRKN and LRRK2 mutations had marked or satisfactory responses after DBS, whereas GBA patients showed variable results in motor symptoms, varying from marked or satisfactory improvement to unsatisfactory responses. In general, the overall benefit of DBS for SNCA, GBA and LRRK2 mutations may be decreased due to the rapid progression of cognitive and neuropsychiatric symptoms. In cases with PRKN, mutations and unsatisfactory responses after DBS, unsatisfactory levodopa responses before DBS, more advanced axial symptoms at a relatively early disease stage and target selection could be some reasons [13,51,53]. The benefit of DBS might also be restricted to a subgroup of LRRK2 patients, depending on the type of mutation they carry. G2019S LRRK2 carriers are three times more susceptible to early-onset dystonia compared to idiopathic PD patients, and DBS appears be beneficial for them. On the contrary, R1441G mutation carriers have shown worse DBS outcomes compared to non-carriers. p.T2031S and p.N1437H (c.4309A > C) mutation carriers have also been observed to have less favorable outcomes; however, these findings are limited by the small number of reported DBS-treated cases of rarer LRRK2 mutations. Restricted data regarding PINK1 and VPS35 do not allow definite conclusions, either. Different outcomes associated with different mutations were carefully assessed in a recent study of 20 patients with a follow-up up to 2 years after DBS. Significant motor improvements were reported in the PRKN, LRRK2 and GBA groups, with better results in the PRKN group compared with the LRRK2 and GBA groups—a result which could be largely attributed to the inclusion of R144G carriers in the LRRK2 group, which are known to have worse DBS outcomes [16].

This review has some limitations. An increased number of studies are case reports or case series or retrospective, short-term studies. Overall, the number of patients that were examined is restricted and the methodology used among studies is heterogeneous, not allowing us to draw definite conclusions. Poorer quality studies have a tendency for bias. Publication bias is also a limitation. The majority of studies examine the effect of STN-DBS, whereas there are scarce data on the effect of GPi- or Vim-DBS on PD patients with genetic mutations. Moreover, most studies also focus on the effect of DBS on motor symptoms, whereas non-motor symptoms are not thoroughly addressed.

Future studies with increased numbers of patients and longer follow-ups and examining the effect of DBS on not only motor but also non-motor symptoms of the disease, such as cognitive function, are awaited to highlight the importance of DBS in PD management. The study of sympathetic nervous system activity with the use of advanced signal analysis of electrodermal activity could be used as an additional biomarker of PD non-motor symptoms in future studies [65,66]. The impact of different genetic mutations on DBS outcome should be also addressed in different populations, as well as possible side-effects. Importantly DBS has also been found to promote gene expression, induce alternative splicing, affect methylation patterns and activate specific signaling pathways [67,68]. Additional studies further examining these issues will increase our understanding of the mechanisms and pathways through which DBS can exert its beneficial effect.

## 5. Conclusions

In summary, DBS appears to have a beneficial effect on motor symptomatology in the shorter term in patients with PRKN, GBA and LRRK2 (non-R144G) mutations, as their motor function and motor fluctuation has been found to be improved after surgery. Patients with GBA mutations may be associated with less sustained motor improvement at the intermediate follow-up or increased frequency of non-motor symptoms after DBS at the long-term follow-up, especially due to cognitive impairment. The type of mutation is also very important, and a characteristic example is LRRK2. In general, LRRK2 mutation carriers respond well to DBS surgery; however, PD patients carrying the R1441G mutation have been observed to have a worse response after DBS surgery. Limited data support that PINK1 mutation carriers show a transient therapeutic benefit after surgery; however, they developed axial symptoms and dyskinesia, and were almost not responsive to drug therapy and neurostimulation. Thus, the presence of genetic mutations can affect DBS outcomes in PD patients, and it may be useful to incorporate genetic data in the management of PD patients; however, additional larger, multicenter, long follow-up studies are needed in order to clarify the impact of genetics on DBS outcomes, especially for rarer genetic causes of the disease.

## Figures and Tables

**Table 1:** The GBA gene and DBS outcomes of PD patients.

Gene	Study Design	N	Type of DBS	Follow-up	Main Results	Reference
GBA	Case series	9	STN-DBS	6–10 yrs	GBA carriers—increase in axial motor impairment, decline in therapeutic efficacy and cognitive decline	[12]
	Case series	94	STN-DBS GPi-DBS Vim-DBS	1–5 yrs	GBA carriers—steeper cognitive decline	[13]
	Case–control	34	STN-DBS GPi-DBS	7.5 yrs	GBA carriers—more severe cognitive impairment, non-motor symptoms and lower quality of life	[14]
	Retrospective	208	STN-DBS	1 yr	GBA carriers—earlier cognitive decline	[15]
	Systematic review	19	STN-DBS	2–6 yrs	GBA carriers—faster cognitive and functional decline po	[16]
	Systematic review and meta-analysis	33	STN-DBS	2–7 yrs	GBA carriers—worse post-surgical cognitive and functional performance	[17]
	Case	1	STN-DBS	4 yrs	c.348_349delCA, p. Tyr116* LRP10 and p. Leu444Pro GBA—improvement in motor function, but rapid cognitive deterioration	[18]
	Case report	1	STN-DBS	14 yrs	G325R GBA mutation—stable cognitive and neurobehavioral performances	[19]
	Case report	1	STN-DBS	3 yrs	Improvement in motor functions and quality of life after DBS with no cognitive decline	[20]
	Case–control	366	STN-DBS	3–5 yrs	GBA carriers—more rapid cognitive decline	[21]
	Cross-sectional	66	STN-DBS	1 yr	GBA1 + DBS+ PD—more severe cognitive dysfunction	[22]
	Retrospective	365	STN-DBS	1–5 yrs	GBA carriers—significant motor improvement, reduction in fluctuations, dyskinesias and impulsive-compulsive disorders, faster rate of cognitive decline	[23]
	Meta-analysis	380	STN-DBS		GBA carriers—more prone to cognitive decline po and a low quality of life	[24]

N: number of individuals studied; DBS: deep brain stimulation; STN: subthalamic nucleus; GPi: globus pallidus internus; Vim: ventralis intermediate nucleus; yrs: years; po: postoperatively.

**Table 2:** The SNCA gene and DBS outcomes of PD patients.

Gene	Study Design	N	Type of DBS	Follow-Up	Main Results	Reference
SNCA	Case report	1	STN-DBS	4 yrs	Improvement in motor fluctuation, reduction in the dose of anti-parkinsonian drugs	[26]
	Case report	1	STN-DBS	2 yrs	Short-term follow-up—reduction in UPDRS III, long-term—cognitive decline	[28]
	Case report	1	GPi-DBS	1 month	Improvement in motor features, reduction in the dose of anti-parkinsonian drugs	[29]
	Genetic association study	85	STN-DBS	2 yrs	rs356220 carriers—better response in UPDRS-III and motor and axial symptomatology	[30]
	Genetic analyses of DBS group vs. BMT group	176	STN-DBS	2 yrs	rs356220—improvement in quality of life	[31]
	Retrospective observational study	4	STN-DBS	3.5 yrs	Significant decrease in the levodopa, p.A53E patients—benefits to motor fluctuation, but in long-term follow-up, progressed axial, cognitive and psychiatric symptoms	[33]

DBS: deep brain stimulation; STN: subthalamic nucleus; GPi: globus pallidus internus; yrs: years; UPDRS: Unified Parkinson’s Disease Rating Scale; BMT: best medical therapy.

**Table 3:** The LRRK2 gene and DBS outcomes of PD patients.

Gene	Study Design	N	Type of DBS	Follow-Up	Main Results	Reference
LRRK2	Cross-sectional study	37	STN-DBS	5 yrs	G2019S: no significant difference po	[36]
	Case series	94	STN-DBS	1–5 yrs	G2019S: no significant difference po	[13]
	Case–control study	39	STN-DBS	6–12 months + 3 yrs	G2019S: no significant difference po	[38]
	Case–control study	97	STN-DBS	1 yr	G2019S: no significant difference po	[37]
	Case–control study	69	STN-DBS	9–10 yrs	G2019S patients—sustained beneficial effect T2031S patient—not favorable outcomes	[39]
	Genetic association study of common SNPS versus DBS response	85	STN-DBS	2 yrs	rs1491923 LRRK2—did not predict motor symptom progression after STN-DBS	[30]
	Retrospective case–control study	103	STN-DBS STN + GPi-DBS	Mean po follow-up 7.0 ± 4.1 yrs	LRRK2—no significant differences in motor outcomes po	[40]
	Case–control study	27	STN-DBS	2 yrs	G2019S: better surgical response	[41]
	Descriptive case report	1	STN-DBS	3 months	G2019S: good motor response, levodopa-related dystonia	[42]
	Systematic review and meta-analysis	33	STN-DBS	Mean follow-up of 12 months	LRRK2 mutation carriers—sustained improvements in UPDRS-IV	[17]
	Retrospective cohort, case–control study	87	STN-DBS, GPi-DBS	2 yrs	G2019S: slightly slower rate of motor progression	[43]
	Case report	1	STN-DBS	8 yrs	Y1699C or R793M mutation carriers—sustained improvement in UPDRS	[44]
	Descriptive case report	1		2.5 yrs	Marked improvement in UPDRS Part III scores in the “on” and “off” states	[45]
	Case series	45	STN-DBS	6 months	R1441G carriers—worse response	[46]
	Systematic review	19	STN-DBS	2–6 yrs	R1441G carriers—worse response DBS	[16]
	Case report	1	STN-DBS	2 yrs	Double LRRK2 R1441G and G2385R mutation—severe motor psychiatric complications 1 yr po	[47]
	Retrospective case–control study	57	STN-DBS	1 yr	G2385R mutation carriers—no significant difference on motor outcomes except in terms of rigidity	[48]

N: number of individuals studied; DBS: deep brain stimulation; STN: subthalamic nucleus; GPi: globus pallidus internus; yrs: years; po: postoperatively.

**Table 4:** The Parkin gene and DBS outcomes of PD patients.

Gene	Study Design	N	Type of DBS	Follow-Up	Main Results	Reference
Parkin	Case–control study	54	STN-DBS	1–2 yrs	Levodopa doses significantly lower in patients with two parkin mutations	[50]
	Case series	94	STN-DBS GPi-DBS	1–5 yrs	Similar clinical outcomes po	[13]
	Cross-sectional study	37	STN-DBS	5 yrs	Similar clinical outcomes po	[36]
	Case–control study	9	STN-DBS	2–5 yrs	Similar clinical outcomes po	[52]
	Prospective observational study	80	STN-DBS	Short-term: 3–12 months Long-term: 3–6 yrs	In the long-term follow-up: similar clinical outcomes	[51]
	Case–control study	36	STN-DBS	21.6 ± 13.1 months	Similar clinical outcomes	[53]
	Case report	1	STN-DBS	1 yr	Significant motor improvement, reduction in the levodopa-equivalent daily dose, complete resolution of severe dyskinesias	[54]
	Case report	1	STN-DBS	8 months	Eminent improvement in PD symptoms po	[55]
	Case report	1	STN-DBS	6 months	Improvement of the severity of motor fluctuations and dyskinesia, reduced doses of anti-parkinsonian drugs, stable cognitive performance	[56]
	Case report	1	STN-DBS	Not reported	Improvement of UPDRS part III scores, reduced doses of anti-parkinsonian drugs	[57]
	Case report	1	STN-DBS	15 yrs	Significant improvement in motor symptoms and fluctuations	[58]
	Case report	1	STN-DBS	Not reported	Homozygous c.G859A parkin mutation—eminent clinical improvement po but the patient soon reported more “off” periods with painful dystonia during the luteal phase of her menstrual cycle	[59]

N: number of individuals studied; DBS: deep brain stimulation; STN: subthalamic nucleus; GPi: globus pallidus internus; po: postoperatively.

**Table 5:** The PINK1 gene and DBS outcomes of PD patients.

Gene	Study Design	N	Type of DBS	Follow-Up	Main Results	Reference
PINK1	Case report	1	STN-DBS	3 yrs	Dyskinesias, freezing of gait and a sub-continuous tremor; however, good control of motor fluctuations and better quality of life po	[60]
	Prospective observational study	80	STN-DBS	Short-term: 3–12 months Long-term: 3–6 yrs	In the long-term follow-up: similar clinical outcomes	[51]
	Case report	1	GPi DBS	Short-term: 1 + 2 months, long-term: >4 yrs	After a transient benefit, the patient in the long-term follow-up—not responsive to medical therapy and stimulation, unable to walk, deterioration of dystonia	[61,62]

N: number of individuals studied; DBS: deep brain stimulation; STN: subthalamic nucleus; GPi: globus pallidus internus; yrs: years; po: postoperatively.

**Table 6:** The VPS35 gene and DBS outcomes of PD patients.

Gene	Study Design	N	Type of DBS	Follow-up	Main Results	Reference
VPS35	Descriptive case series	2	STN-DBS	1 and 8 yrs	D620N mutation carriers— good po response	[63]
	Descriptive case report	1	STN-DBS	5 yrs	Marked improvement in motor symptoms	[64]

N: number of individuals studied; DBS: deep brain stimulation; STN: subthalamic nucleus; yrs: years; po: postoperatively.

## Data Availability

No new data were created or analyzed in this study. Data sharing is not applicable to this article.
